# Combined ergonomic exposures and development of musculoskeletal pain in the general working population: A prospective cohort study

**DOI:** 10.5271/sjweh.3954

**Published:** 2021-04-27

**Authors:** Lars L Andersen, Jonas Vinstrup, Emil Sundstrup, Sebastian V Skovlund, Ebbe Villadsen, Sannie V Thorsen

**Affiliations:** National Research Centre for the Working Environment, Copenhagen, Denmark; Sport Sciences, Department of Health Science and Technology, Aalborg University, Aalborg, Denmark

**Keywords:** back pain, musculoskeletal disorder, neck pain, occupational exposure, physical workload, shoulder pain

## Abstract

**Objective:**

This study aimed to investigate the importance of combined ergonomic exposures at work for the development of musculoskeletal pain.

**Methods:**

Through four rounds (2012–2018) of the Work Environment and Health in Denmark Study, 18 905 employees of the general working population replied to a baseline and 2-year follow-up questionnaire. First, a k-means cluster analysis of seven ergonomic factors (back bending, arm above shoulders, lifting etc., from ‘never’ to ‘almost all the time’) identified nine naturally occurring clusters. Second, using a weighted survey regression model controlling for age, gender, survey year, education, lifestyle, influence at work, and pain intensity at baseline, we estimated development of pain intensity (0–10) in the neck-shoulder and low-back in these clusters. The largest cluster served as reference to the other clusters and was characterized by low ergonomic exposures.

**Results:**

Clusters characterized by multiple combined ergonomic exposures for a relatively high percentage of the working time showed the largest increase in neck-shoulder as well as low-back pain intensity from baseline to follow-up. However, clusters characterized by high exposure to a few specific ergonomic factors also increased pain significantly, eg, standing/walking combined with lifting/carrying or twisted/bent back for the majority of the working time increased low-back pain, whereas repetitive arm movements for the majority of the working time with or without standing/walking increased neck-shoulder pain.

**Conclusion:**

Combined occupational ergonomic exposures play an important role in the development of musculoskeletal pain. Workplace preventive approaches should consider this in risk assessments and organization of the work.

The 2017 Global Burden of Disease Study underscores that musculoskeletal disorders remain a global public health burden (1), with low-back pain as the leading cause of years lived with disability (2). Neck pain also remains a serious public health burden, especially in Scandinavian countries (3). In the working population, musculoskeletal pain can have serious consequences for the ability to do the work (4) and increases the risk for long-term sickness (5). While the origin of musculoskeletal pain is multifactorial in terms of interacting biological, psychological and social factors (6), the work environment also plays an important role (7, 8).

Drawing on data from 35 European countries, a recent systematic review from the World Health Organization (WHO) and International Labour Organization (ILO) shows that occupational exposure to ergonomic risk factors remains highly prevalent (9). Thus, a continued effort to identify the most relevant risk factors and practical workplace solutions is crucial. Longitudinal studies have identified several ergonomic risk factors for developing poor health expressed as long-term sickness absence (10–12) and musculoskeletal pain (7, 8, 13–15). Workplace risk assessments often include ergonomic exposure of the low-back, eg, manual lifting, twisting and bending of the back. In terms of musculoskeletal pain, short-term as well as longitudinal studies have documented an exposure–response association between occupational lifting and development of low-back pain (16, 17). A longitudinal study of the general working population in Norway found that prolonged standing, awkward lifting as well as squatting/kneeling were important ergonomic predictors of developing low-back pain (7). Nevertheless, the evidence for a causal association between working posture and low-back pain is not completely clear in systematic reviews, although the majority of studies favors an association (13, 18, 19). Likewise, working with elevated arms is commonly included in workplace risk assessments. A longitudinal study of the general working population found that high physical work demands, neck flexion and awkward lifting were important predictors of neck-shoulder pain (8). Moreover, systematic reviews including mainly longitudinal studies found that working with elbows or hands above shoulder height were associated with development of neck-shoulder pain and disorders (15, 20).

A shared feature of the literature referenced above is the focus on single ergonomic exposures, without considering the many combinations of ergonomic exposures that may occur in real life at the workplaces. From a methodological standpoint, this is understandable as the number of possible combinations increases markedly with the number of exposure variables. For example, six types of exposure each with three response categories result in 729 possible combinations, which would lead to small subgroups, increased risk of random findings, and be next to impossible to interpret in any practical context. An alternative approach – which we use in the present study – is to group individuals using cluster analysis, where the intent is to maximize the variation in ergonomic exposure between groups and minimize the variation within groups. A cluster analysis approach can therefore identify naturally occurring groups of individuals with rather similar types of exposure and may therefore better reflect reality at the workplaces. Thus, clusters are not pre-defined, but determined based on the actual data. This statistical approach is common in the field of machine learning and marketing, eg, to target specific messages to specific groups of consumers, but has been less used in occupational research.

The present study aimed to investigate the importance of combined ergonomic exposures at work for the development of musculoskeletal pain in the low-back and neck-shoulder in the general working population. First, we identified naturally occurring clusters of ergonomic exposures at work. Next, we analyzed the prospective development of pain in these clusters during 2-year follow-up.

## Methods

### Study design and population

This prospective cohort study used all four questionnaire rounds (2012, 2014, 2016, 2018) of the Work Environment and Health in Denmark Study (WEHD) (21, 22). In short, probability samples of workers aged 18–64 years, having an income of ≥DKK3000 (approximately €400) per month during that the last 3 months, and being employed for ≥35 hours per month, were drawn from Danish registers and invited to participate. Through the four rounds, 228 173 invitations were sent of which 127 882 (56%) responses were received. As labor market status could change from the time of drawing the probability sample to the time of replying to the questionnaire, we included only individuals who confirmed on the questionnaire that they were currently employed wage earners (N=110 357). As the WEHD was primarily intended for surveillance of the work environment in Denmark, only a smaller random sample formed part of the cohort that was invited to participate in more than one questionnaire round. Furthermore, only first and second occasion responses for each individual were used, eg, if an individual participated in 2012, 2014 and 2016, only the first two responses were included, ie, the first (2012) as baseline and the second (2014) as follow-up. Thus, 18 905 individuals participated in the cohort and fulfilled the criteria of being wage earners at the time of the questionnaire and replying to the specific questions about ergonomic exposure and pain at baseline and pain at 2-year follow-up. Reporting follows the STROBE guidelines for cohort studies (23).

### Occupational ergonomic factors (exposure)

The questions concerning ergonomic exposure (10, 24) at baseline were: ‘How much of your working time do you… (i) sit (ii) walk or stand? (iii) work with twisted or bent back without support from the hands and arms? (iv) have the arms lifted to or above shoulder height, (v) do the same arm movements several times a minute? (eg, package work, mounting, machine feeding, carving), (vi) squat or kneel when you work? (vii) push or pull? and (viii) lift or carry? The ergonomic exposure variables were checked for multicollinearity (r ≥0.70). The question about sitting at work was strongly and negatively correlated with walking/standing at work (r = -0.89), which makes sense as doing both at the same time is not possible due to the compositional nature of these tasks. The question about sitting was therefore excluded from the cluster analysis. Response options for each question were ‘almost all the time’, ‘approximately 3/4 of the time’, ‘approximately 1/2 of the time’, ‘approximately 1/4 of the time’, ‘seldom / very little’, and ‘never’. To obtain a normalized score, these response categories were recoded to 100, 75, 50, 25, 12½, and 0, respectively, which correspond to percentages of the working time.

### Change in pain intensity (outcome)

Pain intensity in the neck-shoulder and low-back, respectively, was assessed on a horizontal scale of 0–10 as the worst pain experienced during the last three months, where 0 is no pain at all and 10 is worst imaginable pain (25). Participants replied to this at baseline and 2-year follow-up, from which the change-score in pain intensity was calculated. While the distribution of pain intensity at each respective time point had a tail towards higher values (ie, not normally distributed), the change-score followed an almost perfect normal distribution.

### Baseline control variables

Age (continuous variable) and gender for each individual were drawn from the Central Person Register of Denmark. The year of questionnaire reply was entered as a continuous variable. Highest completed education was drawn from a national register (vocational education or less, higher education). Regarding psychosocial work factors we included ‘influence at work’ (two items, normalized on a scale of 0–100, continuous variable) based on the Copenhagen Psychosocial Questionnaire (COPSOQ) (26). Lifestyle included smoking status (categorical variable: daily, once in a while, ex-smoker, never), body mass index (continuous variable, BMI, kg/m^2^), leisure-time physical activity (continuous variable, total weekly hours of leisure physical activity). Pain intensity at baseline (continuous, 0–10) was assessed as described above.

### Occupational groups

We included register-based job codes to describe the distribution of clusters in different occupational groups for descriptive purposes only, and not as a confounder, as the ergonomic factors are somewhat related to the job codes. The Danish version of the International Standard Classification of Occupations (ISCO) [23] provides a six-digit classification, structured as a five-level hierarchical structure based on information from high-quality national registers. The skill requirements in each ISCO group range from I (most basic) to IV (most advanced). Using the first level of the hierarchy, we included the ten available groups: (i) Managers (level III and IV skill requirements), (ii) Professionals (level IV skill requirements), (iii) Technicians and Associate Professionals (level III skill requirements), (iv) Clerical Support Workers (level II skill requirements), (v) Services and Sales Workers (level II skill requirements), (vi) Skilled Agricultural, Forestry and Fishery Workers (level II skill requirements), (vii) Craft and Related Trades Workers (level II skill requirements), (viii) Plant and Machine Operators and Assemblers (level II skill requirements), (ix) Elementary Occupations (level I skill requirement), and (x) Military (level I, II and IV skill requirements).

### Statistical analyses

Using a k-means cluster analysis (Proc FastClus, SAS version 9.4) of the ergonomic exposure variables at baseline, we identified naturally occurring clusters in the working population. This procedure calculates Euclidean distances between individuals, in this case in a 7-dimensional space, and through an iterative process maximizes variance between clusters while minimizing variance within clusters. Before running the cluster analysis, we checked the exposure variables for multicollinearity (r ≥0.70), which led to the exclusion of one of the variables, sitting at work, as described above. Then, the optimal number of clusters was determined by repeating the fastclus procedure with up to 15 clusters and plotting the cubic clustering criterion (CCC), pseudo F, and explained variance (R^2^) against the number of clusters. This showed local peaks in CCC at 7 and 9 clusters with values of 246.8 and 247.1, respectively, which indicates possible good clustering. The pseudo F values were high at both 7 clusters (pseudo F = 6625) and 9 clusters (pseudo F = 5839). The R^2^ value was slightly higher at 9 clusters (R^2^=0.71) than at 7 clusters (R^2^=0.68). Thus, we chose to use the model with 9 clusters for further analyses. Exposure estimates within clusters are reported as mean values for each separate ergonomic factor.

Using a survey regression model (Proc SurveyReg, SAS version 9.4), which incorporates the sample design into the analyses, we modelled the change in pain from baseline to follow-up in the identified clusters. Separate analyses were performed with neck-shoulder and low-back pain intensity, respectively, as outcomes. Cluster was the predictor variable (9 categories). The largest cluster – characterized by low exposure to the ergonomic factors – served as the reference cluster. Control variables included age, gender, education, year of questionnaire, BMI, smoking, leisure time physical activity, influence at work, and pain intensity at baseline. To ensure that the estimates were representative for wage earners in Denmark, each individual was assigned a weight value (model-assisted weights) based on information from high-quality national registers (gender, age, occupational industry, highest completed education, family income, family type and origin). Missing data were not imputed as the weight variable repairs both non-response and deviations of the probability sample from the population. For the change-score in pain intensity from baseline to follow-up, results are reported as differences of least-square means and 95% confidence intervals (CI) (ie, differences over time compared with the reference cluster).

## Results

Table 1 shows the descriptive baseline characteristics of the 18 905 included participants in terms of age, gender, education, lifestyle, influence at work, and musculoskeletal pain. Pain intensity at baseline was 2.7 [standard deviation (SD) 2.8] and 2.4 (SD 2.8) in the neck-shoulder and low-back, respectively. Table 1 does not show the baseline values of each individual cluster, but for the continuous variables they differed only slightly in terms of age (mean range 44.5–47.9 years), BMI (mean range 25.4–26.3), leisure time physical activity (mean range 4.9–5.5 hours) and influence at work (mean range 72.4–82.2). For pain intensity in the neck-shoulder (means from cluster 1–9: 5.0, 4.6, 3.6, 3.8, 3.7, 3.7, 2.6, 2.6, 2.1, respectively) and low-back (means from cluster 1–9: 4.9, 3.9, 3.6, 3.9, 2.9, 2.9, 2.3, 2.4, 1.7, respectively), the differences between clusters were somewhat larger, underscoring the importance of adjusting for baseline pain intensity.

**Table:** Baseline characteristics of the participants. Values are percentage of participants or mean and standard deviations (SD).

	N	%	Mean	SD
	
Questionnaire round				
2012–2014	9093	48.1		
2014–2016	653	3.5		
2016–2018	9159	48.5		
Age (years)	18 905		46.7	9.7
Gender				
Men	8629	45.64		
Women	10 276	54.36		
Highest education attained				
Vocational education or less	9810	52.2		
Higher education	8986	47.8		
BMI (kg/m^2^)		18 731	25.7	4.4
Physical activity during leisure (hours per week)	18 830		5.2	3.1
Smoking				
Yes, daily	2567	13.6		
Yes, once in a while	921	4.9		
Ex-smoker	5628	29.9		
No, never	9708	51.6		
Influence at work (0–100)	18 878		79.3	18.5
Pain intensity (0–10)				
Neck-shoulder	18 805		2.7	2.8
Low-back	18 816		2.4	2.8

Table 2 shows the fully adjusted and weighted estimates for the development in neck-shoulder and low-back pain intensity from baseline to 2-year follow-up in the identified clusters compared with the reference cluster (cluster 9, low physical work demands). The mean percentage of working time exposed to each of the seven ergonomic factors in each cluster is visualized using color-intensities, ie, higher intensity of the color red signifies higher mean ergonomic exposure in that cluster. Cluster 1 and 2 were characterized by several combined ergonomic exposures for a relatively high percentage of the working time and showed the largest increase in neck-shoulder and low-back pain intensity. Some of the clusters characterized by a few specific exposures for a high percentage of the working were also relevant; cluster 3 and 4 were characterized by standing/walking combined with lifting/carrying (cluster 3) or working with the back twisted/bent (cluster 4) for about two-thirds of the working time and showed a significant increase in low-back pain. Cluster 5 and 6 were characterized by repetitive arm movements for about three quarter of the working time combined with prolonged standing/walking (cluster 5) and little standing/walking (cluster 6). Both clusters showed a significant increase in neck-shoulder pain. Cluster 7 and 8 were characterized by standing/walking for about half of the time (cluster 7) and almost all the time (cluster 8), respectively, while having low exposure to the other ergonomic factors. Both clusters showed a small, but statistically significant, increase in low-back pain.

**Table 2:** Colour-intensity map of combined ergonomic exposures (mean percentage of working time) and changes in neck-shoulder and low-back pain intensity [95% confidence interval (CI)] from baseline to 2-year follow-up in the identified clusters (C) compared with the reference cluster (cluster 9, low physical work demands). N and % are number of participants and percentage of the total number of participants, respectively, for each cluster.

C	N	%	Ergonomic factors (percentage of working time)						Differences of LS means for the change in pain intensity from baseline to 2-year follow-up (95% CI) ^a^		

			Walking, standing	Arms above shoulder	Repetitive arm movement	Back twisted, bent	Lifting, carrying	Pushing, pulling	Kneeling, squatting	Neck-shoulder	Low-back
1	359	1.9	94	67	65	82	82	76	51	0.70 (0.38–1.02)	0.95 (0.59–1.30)
2	423	2.2	90	40	83	82	50	25	16	0.70 (0.37–1.03)	0.59 (0.25–0.92)
3	946	5.0	82	24	15	33	65	30	26	0.50 (0.30–0.71)	0.52 (0.30–0.74)
4	923	4.9	80	24	13	66	21	22	20	0.48 (0.27–0.68)	0.52 (0.31–0.74)
5	527	2.8	93	20	72	20	34	26	12	0.43 (0.16–0.69)	0.26 (-0.01–0.53)
6	698	3.7	26	10	74	16	8	5	4	0.42 (0.21–0.63)	0.17 (-0.05–0.39)
7	4381	23.2	45	10	5	11	12	9	8	0.18 (0.08–0.28)	0.23 (0.13–0.33)
8	3912	20.7	86	12	6	13	14	12	12	0.08 (-0.02–0.19)	0.18 (0.07–0.28)
9	6736	35.6	20	3	2	4	3	1	1	ref	ref

a Controlled for age, gender, education, year of questionnaire, BMI, smoking, leisure time physical activity, influence at work, and pain intensity at baseline.

Sensitivity analyses excluding those with moderate-to-high pain intensity (>3) at baseline did not change the overall picture (not shown in the tables). For example, the increase in low-back pain remained highest for cluster 1 and 2 with change-scores of 0.80 (95% CI 0.29–1.31) and 0.80 (95% CI 0.36–1.25), respectively, compared with the reference cluster.

We also tested the interaction between cluster and age, as well as between cluster and gender, for the development of pain in the neck-shoulder and low-back, respectively. As none of these were statistically significant, we did not proceed with any age- or gender-stratified analyses.

Table 3 shows the relative distribution of the nine clusters in each of the ten different occupational groups (ISCO). Workers in ISCO groups 1–4 (typically higher skill requirement and longer education) and 10 (military) mainly belonged to clusters 7 and 9, to some extent to cluster 8, with only a few percentages in clusters 1–6. Workers in ISCO groups 5–9 (typically lower skill requirement and shorter education) were more distributed across the different clusters.

**Table 3:** Colour-intensity map of the relative distribution (column percentages) of the nine clusters (C) in each of the ten different occupational groups (ISCO). Percentages for cells with less than three observations are not shown.

C	1. Managers	2. Professionals	3. Technicians & Associate Professionals	4. Clerical Support Workers	5. Services & Sales Workers	6. Skilled Agri., Forestry & Fishery Workers	7. Craft & Related Trades Workers	8. Plant & Machine Operators & Assemblers	9. Elementary Occupations	10. Military

	N=884	N=6794	N=2720	N=1728	N=2497	N=93	N=1275	N=967	N=1066	N=120
1	0	0	1	4	10	6	5	7	
2	0	1	2	3	5	6	9	10	
3	2	2	1	3	12	10	14	9	13	6
4	0	3	2	1	10	12	12	5	11	3
5	0	1	3	3	11	5	11	11	
6	2	3	6	7	2	8	2	9	2	3
7	25	27	23	18	22	14	18	24	13	47
8	8	22	10	8	35	24	33	15	26	12
9	61	43	56	57	11	8	4	14	6	29

## Discussion

The main finding of this study is that combined occupational ergonomic exposures play an important role in the development of musculoskeletal pain. Specifically, clusters characterized by several combined ergonomic exposures for a relatively high percentage of the working time showed the largest increase in pain intensity from baseline to follow-up. However, clusters characterized by high exposure to a few specific ergonomic factors were also relevant.

First, some reflections on the type of analysis before discussing the results. The present analytic approach grouped individuals into clusters: ie, with exposure to the different ergonomic factors within each cluster as alike as possible and exposure between clusters as different as possible. The advantage of this approach is that it identifies naturally occurring groups of individuals with rather similar types of exposure while limiting the total number of groups. A shortcoming is that it does not isolate the effect of each type of exposure. While the majority of previous studies in this field have already used the latter approach, the present analyses provide an alternative. Thus, discussing similarities and differences between the present and previous findings is relevant for a better overall interpretation of ergonomic risk factors at the workplace.

Workers in clusters 1 and 2 were exposed to multiple combined ergonomic factors and showed the largest increase in neck-shoulder and low-back pain intensity. This concurs with a previous study evaluating the *number* of ergonomic exposures and the risk for long-term sickness absence (10). In that study, a simple count of exposures showed that a higher number of ergonomic exposures increases the risk for long-term sickness absence in an exposure–response manner. However, a simple count of exposures does not reveal anything about the most relevant *combinations* of ergonomic exposure and may therefore be of little practical relevance for the workplaces. The present study elaborates on previous findings by showing that workers in cluster 1 (ie, those exposed to a combination of lifting/carrying, pushing/pulling, working with the back twisted or bent for the majority of the working day while also doing repetitive arm movements, working with arms over the shoulder, and some kneeling or squatting work) experienced the largest increase in low-back pain. Also, both cluster 1 and 2 included repetitive arm movements, working with the arm above shoulder and lifting/carrying and showed the largest increase in neck-shoulder pain. The workers belonging to cluster 1 and 2 were found across ISCO groups 5–9, representing workers with shorter education and lower skill requirements.

Workers in clusters 3 and 4 stood or walked for more than two-thirds of the working time combined with lifting/carrying (cluster 3) or working with the back twisted/bent (cluster 4) for about two-thirds of the working time and showed a significant increase in low-back pain. Regarding single occupational ergonomic exposure, these are probably the most studied. On a day-to-day basis, higher total lifting load – and thus lifting for a larger part of the working time – increases low-back pain the following day (17). In the longer term, Coenen and coworkers (16) found that lifting loads >25 kg and lifting at a frequency of >25 lifts per day increased the annual incidence of low-back pain by about 3–4%. In another prospective study, Sterud and coworkers (7) found that awkward lifting, eg, twisting or bending the back, increased the risk for low-back pain at 3-year follow-up. Thus, the present study corroborates these findings and show that workers in clusters characterized by ergonomic exposures involving the low-back had the largest increase in low-back pain, although the combination with other ergonomic exposures seems to aggravate this to some extent.

Workers in clusters 5 and 6 performed repetitive arm movements for about three-quarters of the working day combined with prolonged standing/walking (cluster 5) and little standing/walking (cluster 6). Both clusters showed a significant increase in neck-shoulder pain. In accordance, a previous study in the general working population found that repetitive arm movement was associated with increased risk of long-term sickness absence (10). Likewise, repetitive shoulder work has also been shown to predict onset of neck-shoulder pain among industrial and service workers (27). In the present study, and concordant with these findings, workers in clusters involving high exposure to repetitive arm movement were more likely to experience increased neck-shoulder pain at follow-up, although this seemed to be aggravated when combined with other ergonomic exposures as in clusters 1 and 2. Interestingly, clusters characterized by lifting/carrying and working with bent/twisted back (clusters 3 and 4) increased neck-shoulder pain to the same magnitude as clusters 5 and 6, which may explain why neck-shoulder pain increased even more in clusters 1 and 2 having these combined exposures.

Lastly, workers in clusters 7 and 8 stood or walked for about half of the time (cluster 7) and almost all the time (cluster 8), respectively, while having low exposure to the other ergonomic factors. In comparison, workers in the reference cluster stood or walked for about one-fifth of the time. Both clusters 7 and 8 showed a small, but statistically significant, increase in low-back pain, suggesting that excessive standing/walking may not be as health-promoting as often suggested, at least not in an occupational setting and in terms of musculoskeletal pain. In accordance with the present finding, a previous study using body-worn technical measurements suggested that long duration of sitting at work is associated with lower intensity of low-back pain among healthcare workers (28). Likewise, a systematic review – mainly including cross-sectional studies – found that substantial occupational standing was associated with higher low-back pain (29). Thus, while using standings desks and breaking up inactivity is often recommended, too much standing should probably be avoided. Providing standing workers with the opportunity for frequent breaks or job rotation, including seated tasks, may be a practical solution.

### Further practical considerations

Within the methodological limitations of an observational study, we would like to consider some further practical aspects. Van der Beek and coworkers (30) provided a structured research framework for developing and implementing workplace interventions preventing musculoskeletal pain. Elaborating on this framework, the results of the present cohort study provide knowledge of the first two steps, including prevalence of and ergonomic risk factors for developing musculoskeletal pain. The third step of the framework considers underlying mechanisms. The underlying mechanisms of the present results may be related to the time-wise accumulation of specific and combined ergonomic exposures. As an example of specific ergonomic exposures, biomechanical studies show that heavy manual lifting and lifting with arms above shoulders are associated with high loads on the low-back and shoulders (31). Subsequent risk of musculoskeletal pain in these body parts may then accumulate with a higher exposure time leading to muscle fatigue with reduced possibility for recovery. As an example of combined ergonomic exposure, exposure to several ergonomic factors for a higher percentage of the working day may create a general fatigue of the body in addition to the specific effects. Thus, the mechanisms of pain development are likely related to an imbalance between both specific and combined ergonomic exposure, relative to the capacity of the worker to adapt to and recover from those exposures between working days. The fourth to sixth steps of the framework consider development, evaluation and implementation of preventive interventions. An interesting observation of the present study is that even within the same ISCO groups – particularly for ISCO groups 5–9 – the ergonomic exposure varies considerably between workers. This underscores that an individual approach to reducing excessive ergonomic exposure is necessary, especially for these particular ISCO groups. An intervention strategy could be to reduce exposure time to multiple ergonomic risk factors. This may be achieved by using appropriate assistive devices (32) or organizing the work better, eg, by job rotation which also includes less physically demanding tasks. However, simply rotating between several physically demanding tasks would probably not be beneficial, as this would result in a high level of combined ergonomic exposure. Thus, adjusting the physical work demands to the capacity of the worker and including light work tasks or frequent rest break that allow for recovery could be a strategy to prevent development of musculoskeletal pain (33), especially among workers exposed to multiple ergonomic risk factors. Another possibility could be to introduce rest days without physical workload, eg, doing other types of work, to allow for a more complete recovery between strenuous days (17). A contrasting strategy could be to introduce physical exercise at the workplace (34). A recent systematic review found moderate evidence for a positive effect of physical exercise at the workplace for reducing musculoskeletal disorders among workers with physically demanding work (34). Importantly, within the domain of physical exercise, there was strong evidence for a positive effect of workplace strength training, entailing a recommendation of implementing strength training at the workplace in order to reduce musculoskeletal disorders among workers with physically demanding work. Thus, strength training at the workplace to increase physical capacity of the worker could build on top of ergonomic efforts to reduce the physical workload. Overall, this could create a better balance between physical work demands and the physical capacity of the worker.

### Limitations and strengths

There are both limitations and strengths to the present study. The cluster approach is exploratory in nature as clusters are identified based on the actual data and therefore *after* data is collected. As a consequence, this excludes *a priory* hypothesis testing. Nevertheless, based on previous findings (10), we expected clusters characterized by high physical work demands to show the largest increase in pain. Another limitation is the use of questionnaires (self-report) to assess ergonomic exposure. Even though the analyses were adjusted for pain at baseline as well as several other possible confounders, misclassification bias of exposure may occur (35), eg, due to workers with high pain at baseline overrating their level of exposure. To minimize the risk of misclassification bias influencing the overall interpretation, we performed a sensitivity analysis excluding workers with moderate to high pain at baseline. The sensitivity analysis – although having less statistical power – did not change the overall picture. Furthermore, cluster did not interact with age or gender in the development of pain, suggesting that the identified clusters are relevant across age groups and for both men and women. Another limitation is that we did not discriminate between walking and standing, as these were included in the same questionnaire item. As walking is generally considered healthy (36), we recommend future studies to include walking and standing as two separate items. Furthermore, future studies should also include technical measurements to objectively quantify the different ergonomic exposures at work. This would allow for compositional data analyses and thereby avoid problems with multicollinearity and imprecision of self-reports. Non-response bias is a common limitation in questionnaire studies, and the response percentage in the present study was about 56%. However, the sample was based on a random sample of the general working population and we used model-assisted weights based on high-quality registers to adjust for eg, non-response. While generalizable to the general working population of Denmark, the results are also relevant in a broader European context. Based on the European Survey of Enterprises on New and Emerging Risks (ESENER) from 2019, ergonomic factors such as ‘Lifting or moving people or heavy loads’, ‘Repetitive hand or arm movements’ and ‘Tiring or painful positions’ are widespread across the EU-27. Furthermore, for these three ergonomic factors, the prevalence on the EU-27 level is highly comparable to the prevalence in Denmark; 51.7% versus 58.5%, 65.3% versus 65.0%, and 31.6% versus 30.1%, respectively (37). Thus, the present findings may be somewhat generalizable to the EU-27, although country-specific analyses should confirm this.

### Concluding remarks

This study showed that combined ergonomic exposures play an important role in the development of musculoskeletal pain in the general working population. Thus, workers exposed to several combined ergonomic factors for a relatively high percentage of the working time showed the largest increase in pain intensity from baseline to follow-up. However, workers exposed to a few specific ergonomic factors also experienced increased pain intensity.

### Data availability statement

The authors encourage collaboration and use of the data by other researchers. Data is stored on the secure server of Statistics Denmark, and researchers interested in using the data for scientific purposes should contact the authors.

### Funding

Professor Lars L Andersen obtained a grant from the The Danish Working Environment Research Fund for this project (Arbejdsmiljøforskningsfonden, grant number 20195100758). The funder had no role in study design, data collection, data analysis, data interpretation, or writing of the paper.

### Conflicts of interest

The authors declare no conflicts of interest
